# Vitamin C-Dependent Uptake of Non-Heme Iron by Enterocytes, Its Impact on Erythropoiesis and Redox Capacity of Human Erythrocytes

**DOI:** 10.3390/antiox13080968

**Published:** 2024-08-09

**Authors:** Xia Pan, Martin Köberle, Mehrdad Ghashghaeinia

**Affiliations:** 1 Physiological Institute, Department of Vegetative and Clinical Physiology, Eberhard Karls University of Tübingen, 72074 Tübingen, Germany; 2 Department of Dermatology and Allergology, School of Medicine and Health, Technical University of Munich, Biedersteinerstr. 29, 80802 München, Germany

**Keywords:** cationic amphiphilic drugs, desipramine, pregnancy, enterocytes, erythrocytes, testosterone, hepcidin, folates, erythropoietin

## Abstract

In the small intestine, nutrients from ingested food are absorbed and broken down by enterocytes, which constitute over 95% of the intestinal epithelium. Enterocytes demonstrate diet- and segment-dependent metabolic flexibility, enabling them to take up large amounts of glutamine and glucose to meet their energy needs and transfer these nutrients into the bloodstream. During glycolysis, ATP, lactate, and H^+^ ions are produced within the enterocytes. Based on extensive but incomplete glutamine oxidation large amounts of alanine or lactate are produced. Lactate, in turn, promotes hypoxia-inducible factor-1α (Hif-1α) activation and Hif-1α-dependent transcription of various proton channels and exchangers, which extrude cytoplasmic H^+^-ions into the intestinal lumen. In parallel, the vitamin C-dependent and duodenal cytochrome b-mediated conversion of ferric iron into ferrous iron progresses. Finally, the generated electrochemical gradient is utilized by the divalent metal transporter 1 for H^+^-coupled uptake of non-heme Fe^2+^-ions. Iron efflux from enterocytes, subsequent binding to the plasma protein transferrin, and systemic distribution supply a wide range of cells with iron, including erythroid precursors essential for erythropoiesis. In this review, we discuss the impact of vitamin C on the redox capacity of human erythrocytes and connect enterocyte function with iron metabolism, highlighting its effects on erythropoiesis.

## 1. Introduction

**Figure 1:**
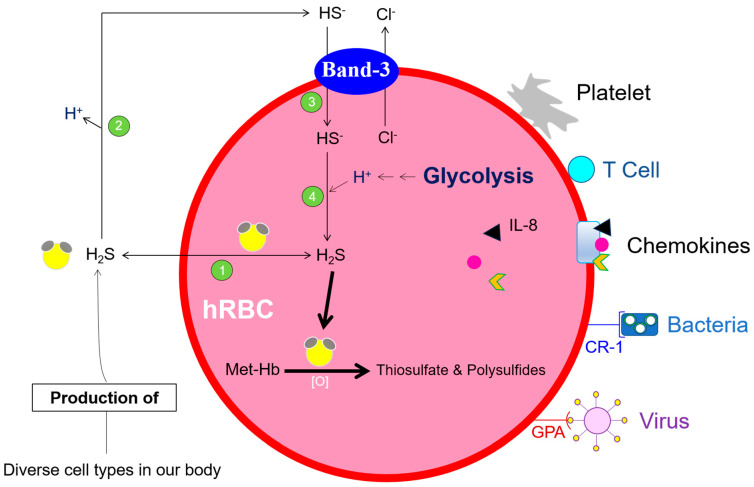
**Transmembrane H_2_S diffusion and Band-3 mediated Cl**^−^**/HS**^−^ **exchange in hRBCs.** Methemoglobin (Hb-Fe^3+^)-mediated H_2_S degradation ensures the maintenance of physiological plasma and tissue concentration of free H_2_S. The Cl^−^/HS^−^/H_2_S cycle is also efficiently involved in net acid (H^+^-ions) efflux; for more details, see the following review [15]. For interactions of hRBCs with endogenous cells and pathogens, see the main text.

A considerable proportion of the cell volume of organelle-free mature human erythrocytes (hRBC) consists of hemoglobin, the molecule responsible for respiratory gas exchange of CO_2_, O_2,_ and CO. Heme is an iron-containing heterocyclic molecule with an iron ion (Fe^2+^) at its center. Iron has the ability to reversibly switch between its two most common oxidation states: ferrous (Fe^2+^) and ferric (Fe^3+^) forms. Both CO and O_2_ solely bind to Fe^2+^, whereas NO binds to both Fe^2+^ and Fe^3+^. Methemoglobin (Hb-Fe^3+^), produced during the auto-oxidation of oxyhemoglobin (HbFe^2+^-O_2_), has an essential physiological function. hRBCs as well as diverse cell types in our body (e.g., hepatocytes and astrocytes), produce the anti-inflammatory signaling molecule hydrogen sulfide (H_2_S) [1,2,3]. hRBCs with their methemoglobin, are significantly involved in the degradation of H_2_S and thus regulate H_2_S levels in blood and tissues [4]. A remarkable property of hydrophobic and cell membrane permeable H_2_S is its carrier- and facilitator-independent transmembrane diffusion [5]. hRBCs are versatile, polyfunctional, and highly complex. They interact with endogenous cells (platelets and lymphocytes) as well as with pathogens (bacteria and viruses). Both complement receptor 1 (CR-1) and glycophorin A (GPA) mediate the attachment of hRBCs to bacteria and viruses [6], respectively, leading to phagocytosis and final elimination of RBCs-bound bacteria and viruses in the liver and spleen [7]. hRBCs also promote the proliferation of activated CD8^+^ T cells [8] and actively absorb infectious HIV-1 virions [9]. Thus, RBCs considerably relieve our immune system. hRBCs can rapidly and reversibly bind an array of chemokines, including IL-8, a leukocyte chemotaxin [10,11,12]. As a result, excessive stimulation of leukocytes and uncontrolled inflammatory responses are avoided. Hemoglobin α and β chains bind LPS (lipopolysaccharide, an endotoxin of Gram-negative bacteria) and neutralize its activity [13,14]. Functioning hRBCs and their production (erythropoiesis), however, require a functioning gastrointestinal tract that makes the components of the ingested food, such as ions and vitamins, available systemically. In this review, we connect vitamin C-dependent uptake of non-heme iron in enterocytes, highlighting their systemic effects on erythropoiesis (Figure 1).

## 2. pH-Dependent Solubility and Uptake of Vitamin C and Dietary Non-Heme Iron

The proper uptake, storage, systemic distribution, and utilization of iron are prerequisites for general health and well-being. For hemoproteins formation (e.g., hemoglobin [16] and ascorbic acid-dependent transmembrane ferrireductases of the cytochrome b_561_ class [17]), various body tissues store iron in the cytosolic protein complex ferritin. Macrophages of the spleen, liver, bone marrow, and skeletal muscle are further prominent storage sites for iron. Regarding one electron transport capacity, iron shows mixed valence states, designated as ferrous (Fe^2+^) and ferric (Fe^3+^) forms. Freely available ferrous iron becomes cytotoxic in the presence of the respiratory by-product hydrogen peroxide (H_2_O_2_) through highly reactive hydroxyl radical formation via Fenton’s reaction [18]. The environment of the digestive tract, especially of the duodenum is complex and adaptable. Following ingestion and upon entry of food into the duodenum, a dual and coincident stimulation of the external secretory functions of the liver and pancreas occurs, i.e., the flow of bile and pancreatic juice into the duodenum. Enterocytes covering the lumen of the intestinal mucosa can absorb ions, nutrients, vitamins, hormones, and water and transfer them to the blood. These cells non-competitively absorb both forms of dietary iron: heme and non-heme iron. Heme, released by hydrolysis of hemoproteins through intraluminal proteases and maintained in a soluble form by globin breakdown products, is absorbed intact by the enterocytes [19,20]. Afterwards, microsomal heme oxygenase-catalyzed heme degradation releases inorganic iron [21]. The latter is then either stored in ferritin molecules or transported to the basolateral membrane of the enterocytes for subsequent release into the blood.

The following equation shows the mucosal heme-splitting activity:

Heme + 3 O_2_ + 3^1/2^ NADPH + 3^1/2^ H^+^ + 7 e^−^ → biliverdin + CO + 3^1/2^ NADP^+^ + 3H_2_O + Fe^2+^.

However, iron intake does not directly reflect iron bioavailability. Uptake by enterocytes of dietary non-heme iron, with ferric iron (Fe^3+^) being the most prevalent, is vitamin C-dependent. Ascorbic acid (AA) pH-dependently exerts both a reducing and a chelating effect on iron salts [22]. Both AA and ferric chloride are totally soluble in the acidic milieu of the stomach. This acidic pH causes the displacement of hydrogen ions from AA to ferric iron, leading to AA-iron chelate formation, which remains in solution over a pH range of 2 to 11. This iron chelate can thus mainly be absorbed at the slightly acidic pH of the duodenum [22,23]. In contrast to iron in heme complex, the uptake of non-heme iron is strongly regulated by dietary constituents.

## 3. Roles of Copper Ion (Cu^+^), Regulatory Proteins, and Vitamin C in Non-Heme Iron Absorption across Human Enterocytes

**Figure 2:**
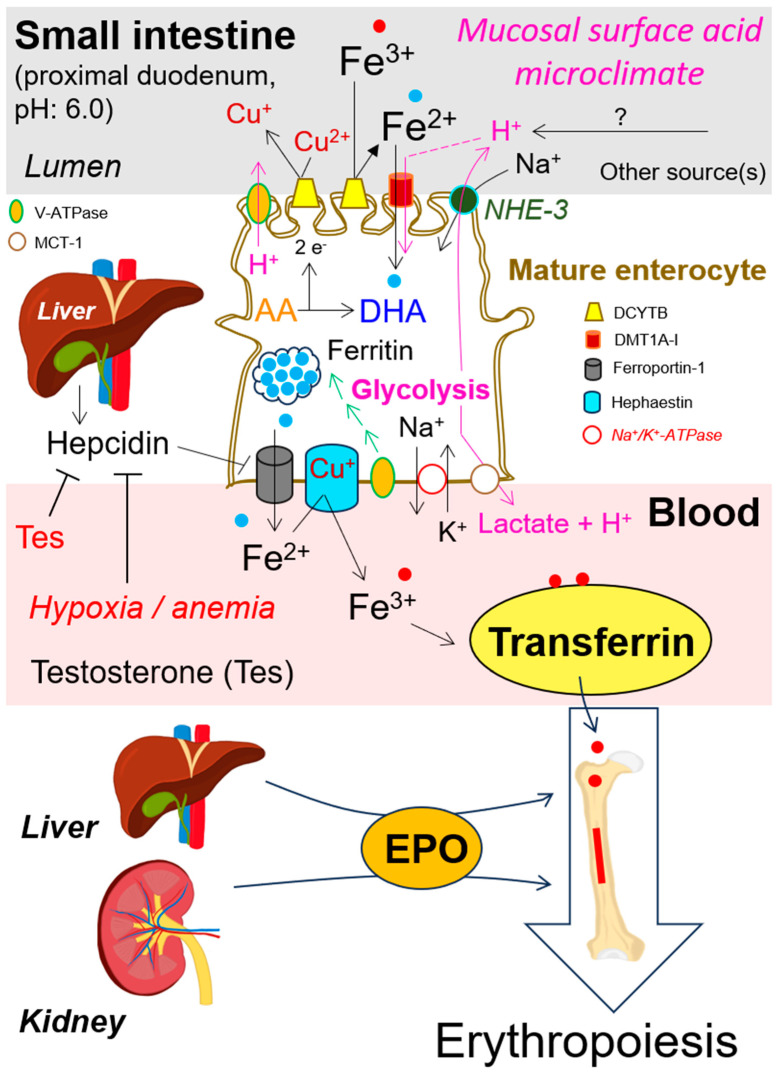
**Uptake and systemic circulation of non-heme iron require several carriers located in the cell membrane of human enterocytes, plasma protein transferrin, and transferrin receptor.** Erythropoiesis requires liver- and kidney-dependent production of erythropoietin.

Two carrier systems accomplish heme-bound iron absorption: (1) heme-carrier protein 1 (HCP-1), a primarily H^+^-coupled folate transporter, and (2) receptor-mediated endocytosis. The apical influx of non-heme iron, especially ferrous iron (Fe^2+^) into the human enterocytes, its basolateral efflux, and re-oxidation to ferric iron (Fe^3+^), engages several regulatory and transporter proteins including their coordinated interactions. A sequence of steps is required prior to loading of monomeric plasma protein transferrin with two ferric iron ions. Both transmembrane proteins duodenal cytochrome B (Dcytb) and divalent metal transporter 1A-I (DMT1), with the former being ascorbic acid (AA)-reducible [17,24,25,26], are highly abundant in the brush-border membrane of duodenal enterocytes. The mammalian di-heme-containing [25,27] and iron-regulated ferric reductase Dcytb reduces ferric iron (Fe^3+^) to ferrous iron (Fe^2+^) prior to its transport by DMT1 [28]. The secondary active transporter DMT1, displays a pH dependence and, in an acidic environment operates as an H^+^/Fe^2+^ cotransporter [29,30,31]. This leads, in the case of enterocytes located at the proximal duodenum (pH 6.0) to rapid intracellular acidification (Figure 2).

## 4. Intracellular Enterocytes’ Lactate Production by Glutamine and Glucose Metabolism

**Figure 3:**
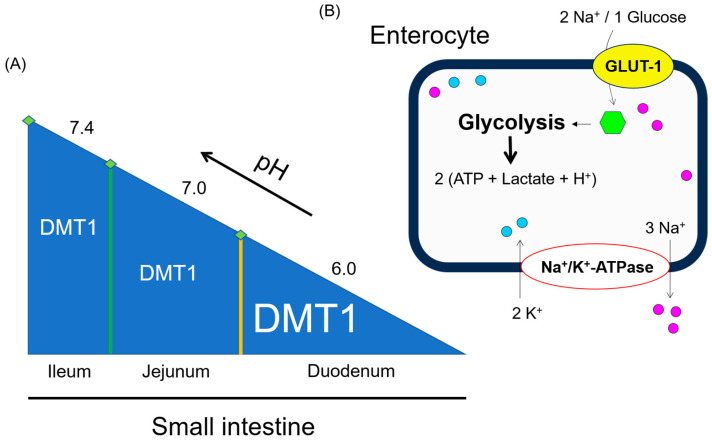
(**A**) **Inverse correlation between DMT1 abundance and pH along the small intestinal. (B) GLUT-1-dependent influx of glucose into human enterocytes.** DMT1: divalent metal transporter 1; GLUT-1: glucose transporter-1.

Enterocytes absorb large amounts of glutamine, glucose, and ketone bodies to cover their energy needs. However, they show substrate preference for oxidative metabolism which can be altered by the availability of other substrates. Both villus and crypt cells have mitochondrial glutaminase activity. Glutamate generated by glutaminase can be transaminated to produce alanine, aspartate, and α-ketoglutarate. The latter an intermediate in the Krebs cycle, contributes to ATP production. The intestinal mucosa consisting of 75% non-lymphoid and 25% lymphoid tissues by mass [32], plays an important role in mucosal immunity [33,34,35,36]. Both cell types—enterocytes and intraepithelial immune cells—residing in this area, utilize glutamine at high and comparable intensity [32,37]. Based on extensive but incomplete glutamine oxidation in the intestinal mucosa, large amounts of glutamine undergo two steps of decarboxylation with the final product being either alanine or lactate depending on the pyruvate pool [38,39]. It is important to mention the gastrointestinal pH profile of healthy subjects. The stomach has a pH of 1.3 to 2. In the small intestine, consisting of three successive sections (duodenum, jejunum, and ileum), the pH values increase. These are about 6.0 in the proximal part of the duodenum, 7.0 at the duodenojejunal junction, and 7.4 in the terminal ileum [40,41]. Figure 3A shows the inverse correlation between DMT1 abundance and pH along the small intestine. Figure 3B illustrates the uptake of glucose in human enterocytes and its utilization by the glycolysis pathway which culminates in the production of two ATP, lactate and H^+^-ions each. Glycolysis is more intense in enterocytes of the proximal than in the distal intestine. Thus, glutamine and glucose metabolism are mainly involved in intracellular enterocyte lactate production.

## 5. Lactate-Induced Activation of Hypoxia-Inducible Factor 1-Alpha (Hif-1α) in Enterocytes and Other Cell Types

Glycolysis, which is independent of intact mitochondrial function, represents a positive selective pressure of evolution for obtaining energy (ATP) within the shortest time and is not restricted to enterocytes. Several healthy cells, e.g., human cytotoxic CD8^+^ T lymphocytes [42] and murine embryonic stem cells [43] temporarily exhibit a significant increase in glycolysis rate during activation and proliferation. This also applies to growing and proliferating tumor cells. However, for the continuation of glycolysis, the extrusion of lactate and H^+^-ions into the extracellular environment is mandatory [44]. This is performed by H^+^-linked monocarboxylate transporters (MCTs) [45]. The generated lactate also activates and stabilizes heme-containing hypoxia-inducible factor 1-α (Hif-1α) [46,47,48], a master regulator of glycolysis and oxygen homeostasis [49]. Hif-1α, discovered by Goldberg et al. [50], in turn, activates the transcription of numerous genes encoding MCT1, MCT4, Na^+^/H^+^-antiporter 1 (NHE-1) [51], vacuolar-type proton pump ATPase (V-ATPase), inducible nitric oxide synthase (i-NOS), heme oxygenase 1 (HO-1), transferrin (Tf), erythropoietin (EPO), ecto-enzyme carbonic anhydrase IX and glucose transporters 1 and 3 (GLUT-1 & -3), of which some are important contributors to erythropoiesis. For lactate-dependent and Hif-1α-mediated control of pH regulating pathways see the following review: [52].

## 6. Hif-1α Mediated Control of pH Regulating Pathways and Their Interplay with Divalent Metal Transporter 1 (DMT1) for Non-Heme Iron Transport

Intracellular pH [pH]_i_ homeostasis is vital to the functioning of cells. Several ion transport mechanisms are involved in this process, e.g., exchangers (NHEs), proton (H^+^) pumps (V-ATPases), and H^+^-MCTs co-transporters, resulting in an alkaline shift in [pH]_i_. As described above in Section 3, DMT1 displays pH dependence and, in an acidic environment (for instance, in the duodenum), operates as an H^+^/Fe^2+^ cotransporter, leading to rapid intracellular acidification of enterocytes. To counteract this alteration of [pH]_i_, reciprocal NHE-3/Na^+^-K^+^-ATPase interplay (concerning Na^+^-ions translocation) as well as the contribution of V-ATPase are needed to apically efflux cytoplasmic H^+^-ions into the intestine lumen. This leads to the generation of an acidic microclimate at the brush border membrane of duodenal enterocytes and the formation of an H^+^-electrochemical gradient. The latter is subsequently used by enterocytes for a DMT1-dependent and H^+^-coupled uptake of Fe^2+^-ions. The most important physiological role of Na^+^-K^+^-ATPase is to channel the free energy of ATP-hydrolysis to intracellularly keep K^+^-ions at high and Na^+^-ions at low concentrations. On the basolateral site of the enterocyte membrane, MCT-1 regulates the equimolar and electroneutral co-extrusion of lactate and H^+^-ions into the circulation. For more details see Figure 2.

## 7. Iron Flux across Enterocytes Membrane, Its Release into the Blood and Distribution by Plasma Transferrin

For iron flux across enterocytes, they require not only apically located influx carriers Dcytb and DMT1 but also basolaterally located transmembrane efflux proteins ferroportin-1 (FPN1) [53] and Cu^1+^-dependent ferroxidase hephaestin [54,55,56,57,58,59,60,61,62]. Fe^2+^ exported by FPN1 is rapidly oxidized back to Fe^3+^ by hephaestin. Subsequently, Fe^3+^-binding plasma protein transferrin (Tf), responsible for systemic iron circulation—Tf-(Fe^3+^)_2_—supplies a wide range of cells with iron, including erythroid precursors essential for erythropoiesis [63,64] and Figure 2.

## 8. Roles of Folates, Vitamin B12, Ferrous Iron (Fe^2+^), Erythropoietin, Testosterone and Hepcidin in Erythropoiesis

The progressive differentiation of short-term hematopoietic stem cells in the bone marrow leads, among other things, to the formation of an erythroid lineage from which terminally differentiated hemoglobin-containing hRBCs arise. This dynamic production process of erythrocytes, referred to as erythropoiesis [65,66,67,68], requires an adequate supply of folates, vitamin B12 (cobalamin), and ferrous iron (Fe^2+^). Deficiency in one or more of these substances results in nutrition-related anemia. Folates are primarily absorbed in the duodenum and jejunum [69,70], whereas intrinsic factor-bound vitamin B12 (vit B12) is mainly absorbed in the terminal ileum [71,72,73]. Stomach acid (which is decreased in subjects with atrophic gastritis), its digestive enzymes (e.g., pepsin), and vit B12-binding glycoproteins haptocorrin and intrinsic factor positively regulate vit B12 absorption [71]. In adults, the kidney serves as the major site (peritubular fibroblasts in the renal cortex) [74,75,76,77], and the liver to a much lesser extent (hepatocytes and perisinusoidal Ito cells) [78,79,80] produces the circulating plasma protein hormone erythropoietin (EPO), the principal regulator of erythropoiesis. For reviews see [81,82,83]. Testosterone suppresses hepcidin [84,85]. The treatment of hypogonadal and especially middle-aged and elderly men with testosterone increases hematocrit levels [86]. This might explain gender-based differences in hematocrit content. The hepatic peptide hormone hepcidin, first discovered by Park et al. 2001 and Pigeon et al. 2001 [87,88], is directly involved in the maintenance of iron homeostasis, and its regulation is tightly controlled at the transcriptional level [89]. Hepcidin synthesis and release from the liver are positively correlated with inflammation and increased plasma and tissue iron levels. Under iron overload, hepcidin binds to its receptor FPN-1, leading to FPN-1 internalization [90,91], ubiquitination, and subsequent lysosomal degradation [91]. Thus, hepcidin-mediated FPN1-downregulation leads to diminished iron efflux, resulting in intracellular iron retention in iron-releasing target cells, e.g., hepatocytes, tissue macrophages, duodenal enterocytes, and placental cells; see also Figure 2. If persistent, this condition impairs iron-dependent erythropoiesis, as systemic iron levels decrease. The following is also of physiological importance: anemia and hypoxia significantly inhibit hepatocellular hepcidin gene expression [92]. Thus, the sophisticated interplay between plasma and tissue iron, testosterone, EPO, hepcidin, anemia, and hypoxia might be understood as a homeostatic loop to maintain the dynamic balance between iron deficiency and overload.

## 9. Glutathione and NADH-Dependent Vitamin C Reduction, Essential Contributors to Maintaining the Redox Capacity of hRBCs

**Figure 4:**
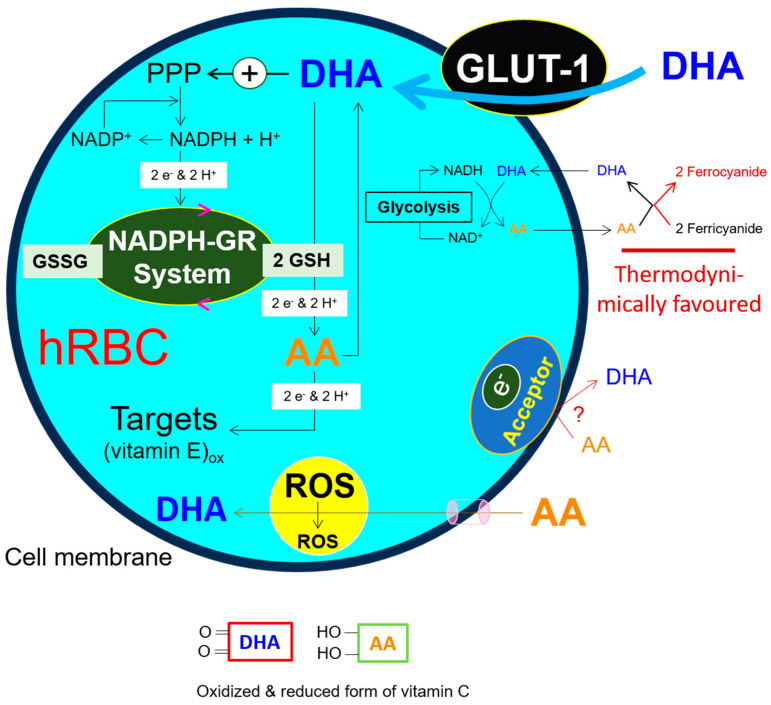
**GLUT-1-mediated influx of oxidized form of vitamin C (DHA) into the mature hRBCs**. The interplay between DHA, PPP, GSH, AA, and the subsequent reduction of vitamin E prevents lipid peroxidation. As a result, cell membrane integrity is maintained and in vivo hemolysis of erythrocytes is minimized. Recently, a link between iron metabolism, lipid peroxidation, and hemolysis was found in stored human and mice erythrocytes [113,114]. Vitamin E is located inside the cell membrane.

In mammals, the plasma concentration of the antioxidant glutathione (GSH), a tripeptide with the structure γ-L-glutamyl-L-cysteinylglycine, is about 25 µM, with typical intracellular concentrations between 1 and 5 mM. The low micromolar plasma concentration of ascorbic acid (AA) is slightly higher than that of GSH, i.e., 40–60 µM. Intraerythrocytic concentrations of GSH and AA are considerably high and amount to 1–2 mM each. AA and DHA uptake are carrier-mediated: the former Na^+^-dependent and the latter Na^+^-independent, carried out by sodium-dependent vitamin C transporters (SVCTs) [93,94,95,96] and members of the glucose-transporter family (GLUT-1, -3 and -4) [94,97,98,99,100], respectively. The driving force for such substrate movements is the protein carrier-mediated secondary active transport with net accumulation of substrate on the other side of the membrane (here: outside → inside direction). Primarily, the mammalian Na^+^/K^+^-ATPase, discovered in 1965 [101], utilizes the glycolytically produced ATP to generate Na^+^/K^+^ asymmetry between cells and their surroundings, i.e., low Na^+^/high K^+^ content within the cell. This gradient is then used to drive diverse secondary active transports [102]. In contrast to nucleated cells, AA is a poor substrate for hRBCs. GLUT-1 transports DHA into hRBCs [103,104,105]. Once within the cells, GSH-dependent two-electron regeneration of AA occurs (DHA + 2 GSH → AA + GSSG), i.e., without involving the monoascorbyl free radical (AFR) intermediate [106,107,108]. This GSH-dependent reduction in DHA is not solely restricted to hRBCs [109]. GSH and DHA are interconnected and form a functional unit. The rapid entry of DHA inflicts on cells a high need for GSH for its reduction to AA. In this context, DHA stimulates the NADPH-generating pentose phosphate pathway (PPP) [110,111]. Subsequently, glutathione reductase (GR) catalyzes NADPH-dependent glutathione disulfide reduction (GSSG + NADPH + H^+^ → 2 GSH + NADP^+^). This self-supporting machinery, as a positive feedback loop, ensures permanent AA regeneration and accumulation within the hRBCs. The subsequent intracellular consumption and extracellular transport of AA (inside → outside direction) and the re-entry of its two-electron oxidized form DHA back into the cells (outside → inside direction) lead to the maintenance of a large intracellular electron pool. This culminates in the vitamin C-dependent high redox capacity of hRBCs; see also Figure 4. NADH generated during glycolysis represents another endogenous source for DHA reduction [112]. The groundbreaking discovery of this group was that a mixture of each mole of AA with 2 moles of ferricyanide instantly resulted in the generation of one mole DHA and two moles of forrocyanide, i.e., without involving the AFR intermediate (AA + 2 ferricyanide → DHA + 2 ferrocyanide) (see Figure 4).

## 10. Impact of V-ATPase on Endosomal pH and DMT1B-II Mediated Iron (Fe^2+^) Release into the Cytosol, and Its Relevance for Erythropoiesis

**Figure 5:**
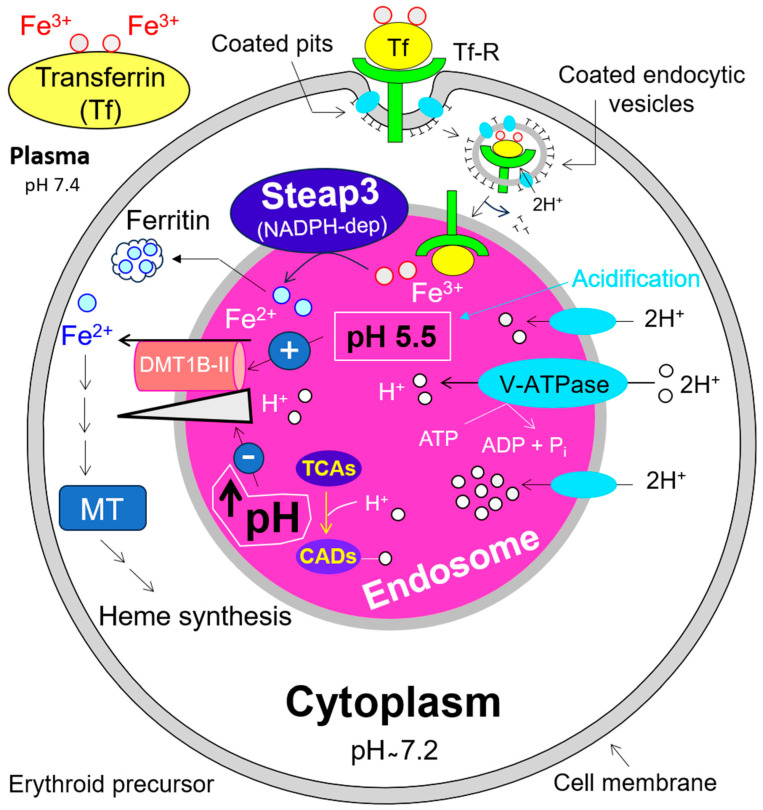
**Iron transport into erythroid precursors**. This process comprises the endocytosis of Transferrin-bound iron, DMT1B-II-mediated Fe^2+^ export from acidified endosomes into the cytoplasm. MT: mitochondria.

In previous sections, we summarized the apical uptake and basolateral efflux of iron across the cell membrane of enterocytes. Now, we will address its subsequent distribution into the blood and utilization in different target cells using the example of erythroid precursors. With almost 30 trillion eukaryotic cells in our body, RBCs, with ~25 trillion, represent nearly 84% of the total cells [115]. Circulating hRBCs have a lifespan of approximately 120 days [116]. Daily, about 1% (~250 billion) of senescent hRBCs are engulfed and degraded by macrophages and replaced to the same extent through erythropoiesis, i.e., two million RBCs are produced per second. A single intact hRBC contains over 270 million hemoglobin molecules. Hemoglobin (Hb) is an iron-porphyrin protein complex consisting of four polypeptide chains, each having a heme prosthetic group with a ferrous iron (Fe^2+^) at its center. Thus, a single hRBC possesses 1.1 billion heme groups or 1.1 billion Fe^2+^ ions. In other words, humans acquire the major part of body iron by catabolizing Hb obtained from senescent RBCs. Dietary iron absorbed in the small intestine and excess iron stored within ferritin in the liver hepatocytes are also available to most body cells. The diferric transferrin—Tf-(Fe^3+^)_2_—is the key iron transport machinery for heme biosynthesis in erythroid precursors. It binds to the transferrin receptor (Tf-R), which clusters in specialized areas of the cell surface, called ‘coated pits’. Coated pits, whose assembly is potassium dependent [117], are pooled into the cytosol to rapidly form coated endocytic vesicles. The latter lose the majority of their coat proteins and are then referred to as primary endosomes (Figure 5). Membrane-embedded V-ATPases are ATP-dependent proton (H^+^)-pumps. They are present in both endomembrane organelles and cell membrane and lead to alkalinization of the cytoplasm associated with acidification of both extracellular milieu (Figure 2) and intracellular compartments, e.g., endosomal and lysosomal lumen [118,119,120] (for details, see Figure 5). In the acidified endosome, ferric iron (Fe^3+^) readily dissociates from Tf [121,122] and is subsequently reduced to ferrous iron (Fe^2+^) by NADPH-dependent endosomal ferrireductase Steap3 (six-transmembrane epithelial antigen of the prostate) [123], prior to its transport into the cytosol by H^+^-coupled endosomal DMT1B-II [124] (see also Figure 5). Cytosolic iron (Fe^2+^) is the essential substrate for heme biosynthesis in erythroid precursors and erythropoiesis. Heme itself controls the synthesis of globin chains—needed for hemoglobin synthesis—at both transcriptional and translational levels. It is important to note that genetic ablation of Steap3 leads to severe hypochromic [125] and microcytic anemia [126].

## 11. Disruption of Lysosomal pH by Tricyclic Antidepressant Desipramine, Its Possible Negative Effect on Endosomal pH, Iron Supply and Erythropoiesis: A High-Risk Drug during Pregnancy?

Tricyclic antidepressants (TCAs) influence norepinephrine (NE) and serotonin (SER) transporters [127]. Desipramine, a representative of TCAs, has two primary targets. On one hand, it preferentially interacts with the NE-transporter and increases NE synaptic transmission by inhibiting NE reuptake, thereby relieving depressive symptoms [128,129,130,131]. On the other hand, desipramine has a direct inhibitory effect on lysosomal acid ceramidase [132] and acid sphingomyelinase [133,134]. Both acid ceramidase [135,136,137,138] and acid sphingomyelinase [139,140,141] are aberrantly over-expressed and highly active in patients with dysregulated sphingolipid metabolism. Under acidic conditions, e.g., in lysosomes, endosomes, or in the cytoplasm of glycolytically active cells, TCAs act as cationic amphiphilic drugs (CADs) (TCAs + H^+^ → CADs), their reactions resembling the formation of ammonium from ammonia and a proton (NH_3_ + H^+^ → NH_4_^+^). The secondary amine and basic lipophilic drug desipramine follow the same principle: it acts as a proton (H^+^-ion) acceptor, depleting the free proton and thus increasing the intracellular pH. Elojeimy et al. (2006) showed that in cancer cell lines, desipramine, even at a relatively low dose of 5 µM, neutralizes lysosomal pH [132]. By the same principle, desipramine and other CADs like imipramine, amitriptyline, chlorpromazine, and chloroquine would be able to neutralize the luminal acidification of endosomes in glycolytically active erythroid precursors. Consequently, H^+^-coupled Fe^2+^ transport into the cytosol of these cells, and thus the proper heme biosynthesis and heme-dependent erythropoiesis, might be severely affected (see Figure 5).

In addition to this, desipramine inhibits NHE-1 activity [142], a major regulator of intracellular pH. As shown in Figure 2, NHE-3 plays an essential role in H^+^-coupled Fe^2+^ transport by DMT1 in enterocytes. To avoid anemia and preserve the naturally increased erythropoietic activity during pregnancy, pregnant women suffering from depression should avoid medications with CAD properties if possible. However, this does not diminish the importance of CAD’s clinical applicability regarding cancer [143,144,145] and anti-viral [146,147] therapies. It is to be noted that the above-mentioned low-dose concentration of desipramine (5 µM) has no significant detectable biological effects on cell organelle-free mature hRBCs [148].

## Data Availability

Not applicable.
